# IL-2 analogues: novel agents circumventing the expansion of T regulatory cells while promoting NK cell activation during IL-2 therapy

**DOI:** 10.1186/2051-1426-1-S1-P89

**Published:** 2013-11-07

**Authors:** Geok Choo Sim, Elizabeth Grimm, ZhiMin Dai, Willem Overwijk, Patrick Hwu, Laszlo Radvanyi

**Affiliations:** 1 Melanoma Medical Oncology, UT MD Anderson Cancer Center, Houston, TX, USA

## 

High dose (HD) IL-2 treatment is a potent form of immunotherapy that can induce durable complete responses in a fraction of metastatic melanoma patients. Its initial purpose was to activate the expansion and anti-tumor responses of NK cells. However, the efficacy of HD IL-2 in achieving this has been relatively limited due to two main reasons. First, IL-2 preferentially expands CD4+CD25+Foxp3+ T-regulatory cells (Tregs) constitutively expressing the high affinity IL-2R consisting of CD25 together with the IL-2Rβ and the common gamma chain rather than NK cells and effector T cells that are mostly CD25-negative and express mostly the IL-2Rβγ lower affinity receptor. In particular, we found that a highly suppressive CD25hi Treg subset expressing ICOS expands the most in response to IL-2. Second, HD IL-2 can be highly toxic by causing vascular leak syndrome due to a surge in pro-inflammatory cytokines and by its indirect activation of CD25 expressing endothelial cells facilitating NO release. Here, we characterized whether a mutant form of IL-2 (F42K) that preferentially binds to the lower affinity IL-2Rβγ complex can be an alternative to wild-type (WT) IL-2 therapy. We first tested the effects of equivalent concentrations of WT IL-2 on PBMC from healthy donors and melanoma patients for 6 or 14 days in vitro. F42K-treated PBMC produced much lower pro-inflammatory cytokines such as IL-6, IL-1α and IFN-γ, and VEGF than WT IL-2. Importantly, F42K selectively expanded NK cells with a decreased expansion of ICOS+ Tregs. In comparison to treatment with WT IL-2, F42K also induced increased NK cell activation by up-regulating the NK activation markers NKp30, NKp44 and TIM-3, but not NKG2D, CD226 and inhibitory KIR molecules such as CD158a and CD158b. F42K also enhanced survival of these highly activated NK cells and prevented apoptosis by inducing a higher expression of Bcl2 than WT IL-2. Furthermore, F42K increased NK cell cytotoxicity against breast cancer and melanoma cells than WT IL-2 associated with TRAIL and granulysin up-regulation in both CD56hi and cytotoxic CD16+CD56+ NK cell subsets. Using a hydrodynamic gene therapy (HGT) approach, C57BL/6 mice treated with plasmids encoding F42K had an earlier, more robust and persistent NK cell expansion in vivo with a dramatic reduction in the expansion of CD4+CD25+Foxp3+Tregs than WT IL-2 plasmid therapy. Lastly, we found that F42K was equally able to expand TILs from melanoma patients as WT IL-2. Our results suggest that F42K has great potential as a non-toxic substitute for WT IL-2 as a cytokine therapy for cancer by potently activating NK cells without Treg expansion either as a monotherapy or in combination with other therapies such as adoptive cell therapy.

